# Novel Carboxypeptidase A6 (*CPA6*) Mutations Identified in Patients with Juvenile Myoclonic and Generalized Epilepsy

**DOI:** 10.1371/journal.pone.0123180

**Published:** 2015-04-13

**Authors:** Matthew R. Sapio, Monique Vessaz, Pierre Thomas, Pierre Genton, Lloyd D. Fricker, Annick Salzmann

**Affiliations:** 1 Department of Neuroscience, Albert Einstein College of Medicine, Bronx, NY, United States of America; 2 Department of Genetic Medicine and Laboratory, University Hospitals of Geneva, Geneva, Switzerland; 3 Department of Neurology, University Hospital, Nice, France; 4 Centre Saint Paul, Hôpital Henri Gastaut, Marseille, France; 5 Department of Molecular Pharmacology, Albert Einstein College of Medicine, Bronx, NY, United States of America; 6 Department of Psychiatry, University of Geneva, Geneva, Switzerland; Icahn School of Medicine at Mount Sinai, UNITED STATES

## Abstract

Carboxypeptidase A6 (CPA6) is a peptidase that removes C-terminal hydrophobic amino acids from peptides and proteins. The *CPA6* gene is expressed in the brains of humans and animals, with high levels of expression during development. It is translated with a prodomain (as proCPA6), which is removed before secretion. The active form of CPA6 binds tightly to the extracellular matrix (ECM) where it is thought to function in the processing of peptides and proteins. Mutations in the *CPA6* gene have been identified in patients with temporal lobe epilepsy and febrile seizures. In the present study, we screened for *CPA6* mutations in patients with juvenile myoclonic epilepsy and identified two novel missense mutations: Arg36His and Asn271Ser. Patients harboring these mutations also presented with generalized epilepsy. Neither of the novel mutations was found in a control population. Asn271 is highly conserved in CPA6 and other related metallocarboxypeptidases. Arg36 is present in the prodomain and is not highly conserved. To assess structural consequences of the amino acid substitutions, both mutants were modeled within the predicted structure of the enzyme. To examine the effects of these mutations on enzyme expression and activity, we expressed the mutated enzymes in human embryonic kidney 293T cells. These analyses revealed that Asn271Ser abolished enzymatic activity, while Arg36His led to a ~50% reduction in CPA6 levels in the ECM. Pulse-chase using radio-labeled amino acids was performed to follow secretion. Newly-synthesized CPA6 appeared in the ECM with peak levels between 2-8 hours. There was no major difference in time course between wild-type and mutant forms, although the amount of radiolabeled CPA6 in the ECM was lower for the mutants. Our experiments demonstrate that these mutations in *CPA6* are deleterious and provide further evidence for the involvement of *CPA6* mutations in the predisposition for several types of epilepsy.

## Introduction

Genetic generalized epilepsies (GGE), formerly called idiopathic generalized epilepsy [1], account for 15% to 20% of all epilepsies [2]. The main features of GGE are generalized seizures with known or presumed genetic defects [3]. Seven clinical categories of GGE syndromes have been reported in the literature [2]. Among these seven, juvenile myoclonic epilepsy (JME) accounts for about 18% of GGE and 5% to 10% of all epilepsies [4]. The typical age of onset for JME is between 12 and 18 years [5], with females comprising 60% of affected individuals [4]. The major clinical characteristics of JME include myoclonic jerks without loss of consciousness and generalized tonic-clonic seizures. Some patients also suffer from absence seizures [6]. Seizures generally occur upon waking or soon after [7]. JME is considered a benign form of epilepsy with a good prognosis when treated with antiepileptic drugs [6]. Despite its relatively mild symptoms, JME typically progresses to generalized tonic-clonic seizures which can be severely disruptive, and can be a medical emergency when long-lasting.

To date, nine loci have been linked to JME and named EJM1 through 9, as reported in the Online Mendelian Inheritance in Man (http://www.omim.org/). The genes for five of the EJM loci have been identified and show Mendelian inheritance patterns. Of these, four encode ion-channels: *CACNB4* (calcium channel, voltage-dependent, beta 4 subunit) [8]; *CASR* (calcium-sensing receptor) [9]; *GABRA1* (gamma-aminobutyric acid A receptor, alpha 1) [10]; and *GABRD* (gamma-aminobutyric acid A receptor, delta) [11]. The non-ion channel gene encodes myoclonin 1 (also called *EFHC1* for EF-hand domain (C-terminal) containing 1), which is thought to interact with the R-type voltage-dependent Ca^2+^ channel CA_v_2.3 [12]. The mechanism by which *EFHC1* mutations cause epilepsy is unclear however, as the protein encoded by this gene has been linked to several effects that could damage brain development [13,14]. JME is also considered to be a disorder with complex genetic inheritance that accounts for only 3% of cases as measured by population-based prevalence [15]. Consequently, the etiology of this disorder most likely involves a complex interaction between several genetic risk factors, each with minor effects, and environmental factors [16]. Three susceptibility alleles have been associated with an increased risk of developing JME in *BRD2* (bromodomain containing 2)[17], *Cx-36* (connexin 36)[18], and *ME2* (malic enzyme 2, NAD(+)-dependent, mitochondrial)[19] genes. Moreover, copy-number variations have been identified on chromosomes 15q13.3, 15q11.2 and 16p13.11 in JME patients [20]. Consequently, single nucleotide polymorphism (SNP) alleles, structural genomic variants and also rare and/or *de novo* mutations might be involved in JME [21].

Recently, we reported SNP alleles and missense mutations in the gene encoding carboxypeptidase A6 (CPA6) in patients showing febrile seizures (FS) and temporal lobe epilepsy (TLE) [22,23]. The *CPA6* gene is located on chromosome 8 and is not related to previously reported loci for JME or other types of epilepsy. The *CPA6* promoter is more extensively methylated in patients with focal epilepsy or FS, further supporting a relationship between this gene and seizure disorders [24]. One well-characterized epilepsy gene, *SCN1A* (sodium channel, voltage-gated, type I, alpha subunit), has previously been reported in both generalized and partial epilepsies [25], which gives some precedence for susceptibility genes occurring in different subtypes of epilepsy.

CPA6 is a member of the M14 metallocarboxypeptidase family of enzymes [26] discovered in a search for novel metallocarboxypeptidase genes in 2002 [27]. It is translated as a 50 kDa proenzyme containing a prodomain, which aids in folding and maintains the enzyme in an inactive state while inside the cell. CPA6 is secreted as a 37 kDa mature enzyme lacking the prodomain, and binds to the extracellular matrix (ECM) where it is enzymatically active [28]. Like other members of this sub-family of enzymes, CPA6 cleaves C-terminal amino acids from peptides and proteins. C-terminal proteolytic cleavages are important post-translational modifications that can activate, modulate, or degrade signaling molecules [26,29]. Previous work has shown that CPA6 prefers hydrophobic amino acids, with specificity dependent on both the P1 and P1’ amino acids [30]. The mRNA encoding CPA6 has been detected in developing and adult mouse brain [31], as well as in adult human brain [22]. In adult mouse central nervous system, *Cpa6* mRNA is most strongly expressed in the olfactory bulb, but has also been detected in the hippocampus and other brain regions [31].

Previous studies identified patients with TLE harboring one mutated *CPA6* allele [22,23]. Biochemical analysis of these disease-associated mutant alleles showed that heterozygous individuals with TLE had one allele with either no enzymatic activity or no expression of the mature enzyme bound to the ECM, leading to a complete loss of functional CPA6 for that allele [22,23]. A missense mutation (Ala270Val) in *CPA6* has also been identified in a family with FS and focal epilepsy [22]. In this family, Ala270Val heterozygotes were unaffected, but homozygotes developed seizures. Biochemical analysis of this mutant revealed that protein levels were reduced by approximately 40% in the ECM [22]. Based on the information from previous studies of *CPA6* mutations in epilepsy patients, we hypothesized that loss of function mutations cause an increased susceptibility to epilepsy. In the present study, we characterized two novel mutations found in a population of patients with JME who also presented with generalized seizures.

## Experimental Procedures

### Subjects

JME patients (Table 1) were recruited as described in Guipponi et al [32]. Patients with JME were randomly selected from neurology departments of different hospitals (Grenoble, Lyon, Marseille, Montpellier, Nice, Paris, Strasbourg) independent of the presence or absence of epileptic syndromes in first-degree relatives. Diagnostic evaluation was made according to the classification of the International League Against Epilepsy [33], and the analysis of records (comprising EEGs) from previous hospitalizations. The population of healthy Caucasian controls used in the present study (Table 1) were described previously [23] and were recruited at the University Hospitals of Geneva. Written informed consent was obtained from all participating individuals and the study was approved by the ethical committee of the University Hospitals of Geneva.

**Table 1:** Demographic and clinical description of JME patients and Caucasian controls.

	Male n, (%)	Female n, (%)	Age at inclusion ± SD	Age at onset ± SD	Familial history of epilepsy n, (%)	Resistance to antiepileptic drug n, (%)	Remission n, (%)
JME patients (n = 127)	56 (44.1)	71 (55.9)	26.9±10.1 (NA: 21)	16.0±13.5 (NA: 41)	39 (42.9) (NA: 36)	28 (23.0) NA: 5	25 (46.3) NA: 73
Caucasian control group (n = 242)	171 (70.7)	71 (29.3)	44.8±12.8	-	-	-	-

SD: Standard deviation. NA: number of patients for whom no information is available.

### Mutations and SNP screening

Experimental procedures for extracting genomic DNA from venous blood and for exploring the 11 exons of *CPA6* (NM_020361.4) by high resolution melt were carried out as described previously [22,23].

### Statistical Analysis

Common variants were analyzed as described previously [23]. First, we verified Hardy-Weinberg equilibrium by using a 2 allele goodness-of-fit test. Then differences in allele and genotype frequencies between cases and controls were determined using UNPHASED software (Version 3.0.10) and a Chi-square test [34]. We used p = 0.05 as a threshold for both tests, and no Bonferroni correction was used. This methodology was applied for analyzing rs10957393 (c.133T>C → p.Phe45Leu), rs17853192 (c.518C>G → p.Ser173Cys), and rs17343819 (c.746A>G → p.Asn249Ser).

### Protein Analysis and Modeling

PreproCPA6 protein sequences were accessed from The National Center for Biotechnology Information and aligned using Clustal Omega. Multiple sequence alignments were visualized and analyzed using GeneDoc to assess degree of conservation across vertebrate evolution, and degree of conservation among human CPA subfamily members. To predict functional effects of amino acid substitutions, protein mutations were analyzed using PolyPhen-2 (genetics.bwh.harvard.edu/pph2/), MutPred (mutpred.mutdb.org/) and SNPs&GO (snps-and-go.biocomp.unibo.it/). Functional effects of amino acid substitutions were also addressed by examining *in silico* mutations in Swiss PDB Viewer as described previously [30]. CPA6 protein models were drawn using PyMOL [35].

### Site-directed mutagenesis

Missense mutations were introduced into the pcDNA3.1(–)hCPA6-HA-H6 [28] plasmid using mismatched primers and PfuUltra II Hotstart DNA Polymerase (Agilent, Santa Clara, CA) according to the QuikChange mutagenesis protocol (Stratagene). Resulting cDNAs were confirmed by sequencing, and express full-length human preproCPA6 under the cytomegalovirus promoter with the hemagglutinin (HA) and hexahistidine (H6) tags at the C-terminus. The same template and method was used to generate CPA6 with a C-terminal FLAG tag (CPA6-FLAG).

### Cell culture and biochemical characterization of mutant CPA6

Human embryonic kidney 293T (HEK293T) cells were cultured in Dulbecco’s modified Eagle’s medium (DMEM, Gibco) supplemented with 10% fetal bovine serum (Gibco) and penicillin/streptomycin (Gibco) at 37°C and 5% CO_2_ in 6-well cluster plates. Cells were transfected with Transfectin (Bio-Rad) according to manufacturer’s instructions. At 48h post-transfection, cells were harvested in PBS, centrifuged, resuspended in PBS + 1X sodium dodecyl sulfate polyacrylamide gel electrophoresis (SDS-PAGE) sample buffer, and boiled at 95°C for 5 minutes. Lysates were then vortexed and centrifuged at 13,000g for 3 minutes, and SDS-PAGE was performed on a denaturing polyacrylamide gel, transferred to nitrocellulose, and probed using an anti-HA antibody (Sigma, 1:5000 or Cell Signaling 1:1000) or an anti-α-tubulin antibody (Sigma, 1:5000) followed by an anti-mouse antibody linked to horseradish peroxidase (Cell Signaling, 1:2000). Blots were incubated in enhanced chemiluminescence reagent (Perkin Elmer) and exposed to x-ray films. Dilutions of some samples were run to ensure exposures were within the linear range of the film. ECM samples were washed several times with PBS to remove material from cells. After washing, 1 ml 0.5mM 3-(2-furyl)-acryloyl-Phe-Phe substrate in 150 mM NaCl and 50 mM Tris, pH 7.4 was added to ECM samples and incubated on a rocking platform for 90 minutes at 37°C. Reactions were stopped by removing the buffer containing the 3-(2-furyl)-acryloyl-Phe-Phe substrate and enzyme activity was determined by measuring the absorbance at 336 nm. CPA6, which remains bound to the ECM, was then extracted by addition of 180 μl of hot SDS-PAGE buffer, and analyzed by SDS-PAGE in the same manner as cell lysates. For the Western blots, equal amounts of cell extract were loaded. All protein bands were densitized using ImageJ. Statistical testing was performed using Student’s t test.

For experiments in which CPA6 was detected in the media, 400 μg/ml heparin (Sigma) was added to culture medium at 24h post-transfection and incubated for 24h. The addition of heparin causes CPA6 to be soluble in the media, and is used as a positive control to show that CPA6 can be detected in the media when present.

For experiments in which two plasmids were transfected at the same time, equal amounts of each plasmid were mixed together before combining with transfection reagent. Samples were prepared and analyzed as described above.

### Pulse-chase analysis

HEK293T cells were grown under the same conditions as above, in individual 35mm plates. Cells were incubated in DMEM lacking methionine and cysteine (Sigma) supplemented with 2 mM glutamine, 5% dialyzed fetal bovine serum (Gemini), and penicillin/streptomycin (Gibco) for 30 minutes (starvation media). Starvation media was removed, and cells were washed once in 37°C PBS containing calcium. Cells were then incubated in starvation media containing 0.1 mCi [^35^S]-labeled methionine/cysteine (Easy Tag EXPRESS^35^S, Perkin Elmer) for 20 minutes (pulse). The pulse period was stopped by removal of medium containing radioisotopes, washing, and incubation in DMEM supplemented with 2 mM methionine and cysteine for the indicated chase time. Cells were washed once in ice cold PBS. Cells were removed from the plate by washing 3x with PBS containing 0.1% Triton X-100 followed by 2x washes with PBS to separate cell debris from ECM. ECM proteins were extracted using 90 μl of 2% SDS in PBS, and analyzed by SDS-PAGE as above. No enrichment for CPA6 was performed and no protease inhibitors were used. Gels were fixed in 40% methanol/10% acetic acid/5% glycerol, incubated in Fluoro-Hance (Research Products International) with 5% glycerol, dried, and exposed to x-ray film at -80°C for 5–15 days.

## Results

**Fig 1:**
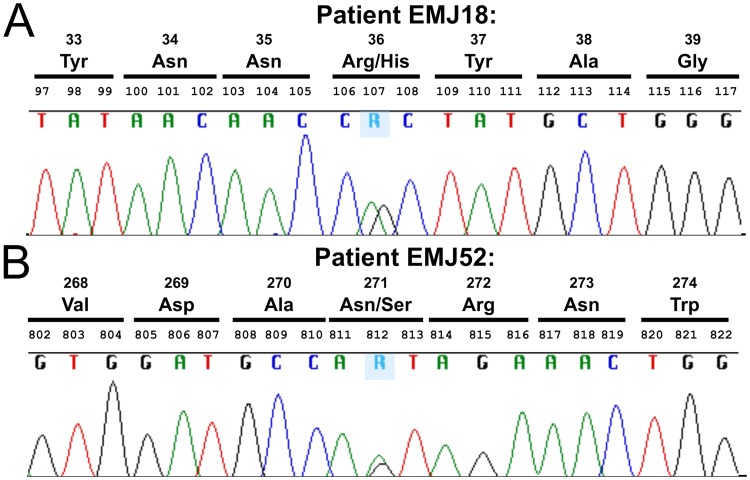
Sequence chromatograms of *CPA6* in two patients showing c.107G>A (Arg36His) and c.812A>G (Asn271Ser). Patients with juvenile myoclonic epilepsy were sequenced to reveal the location of mutations in the *CPA6* gene. (A) Patient EMJ18 showed heterozygosity at base pair 107, as indicated by the presence of both an adenine and guanine peak at the same position (indicated as R). (B) Patient EMJ52 showed overlapping peaks for adenine and guanine at position 812.

Two novel exonic mutations in *CPA6* were found in a population of patients with JME, and were not found in 242 Caucasian controls. Each mutation was found in one individual. The first variant, c.107G>A was uncovered in the heterozygous condition (Fig 1A) in an affected woman (Table 2, subject EMJ 18). This missense mutation affects an arginine residue at position 36, which is replaced by a histidine (Arg36His). The second pathogenic allele was also observed in a JME female (Table 2, subject EMJ 52). She is heterozygous for the genomic variation c.812A>G, which results in a missense mutation (Fig 1B), which changes an asparagine to a serine (Asn271Ser). Because parents of both patients were not available, we could not determine whether these two mutations are *de novo*.

**Table 2:** *CPA6* mutations in JME patients.

Subject	EMJ18	EMJ52
Sex	F	F
Age at inclusion	20	20
Age of onset	16.5	12
Clinical description	Myoclonic seizures, generalized tonic-clonic seizures, absence seizures	Myoclonic seizures, generalized tonic-clonic seizures
*CPA6* mutated allele	c.107 G→A (p.Arg36His) heterozygous	c.812 A→G (p.Asn271Ser) heterozygous
Treatment/resistance to antiepileptic drugs	Valproate/no	Valproate/no
Family history of epilepsy	No	No
Neurological examination	Normal	Normal

We also assessed the frequency of three common SNPs in *CPA6* (rs10957393 Phe45Leu, rs17853192 Ser173Cys, and rs17343819 Asn249Ser) in JME patients compared to healthy controls. First, we verified Hardy-Weinberg equilibrium to test if genotype frequencies in our patient and control populations remained constant through generations [36]. The rs17853192 and rs17343819 genotypic distributions were at Hardy-Weinberg equilibrium among JME patients and controls. The rs10957393 genotypic distributions were under the threshold of significance among JME patients (p = 0.02) and controls (p = 0.001) as found in our previous paper [23]. There was no difference in the genotypic and allelic frequencies of these SNPs in JME patients (S1 Table).

The two mutations found in JME patients were analyzed by PolyPhen-2, MutPred and SNPs&GO to predict the likelihood that they would be deleterious to enzyme function. For Arg36His, PolyPhen-2 reported a HumDiv score of 0.985 (“probably damaging;” sensitivity 0.74; specificity 0.96) and a HumVar score of 0.297 (“benign;” sensitivity 0.86; specificity 0.76). For Asn271Ser, PolyPhen-2 reported a HumDiv score of 1.000 (“probably damaging;” sensitivity 0.00; specificity 1.00) and a HumVar score of 1.000 (“probably damaging;” sensitivity 0.00; specificity 1.00). Using MutPred, the probabilities that Asn271Ser and Arg36His are deleterious mutations were predicted to be 0.819 and 0.485, respectively. SNPs&GO predicted that both mutations would be neutral (reliability index: Arg36His = 4; Asn271Ser = 1). In general the results from prediction algorithms has correlated poorly with experimental evidence [37], necessitating more thorough analysis.

**Fig 2:**
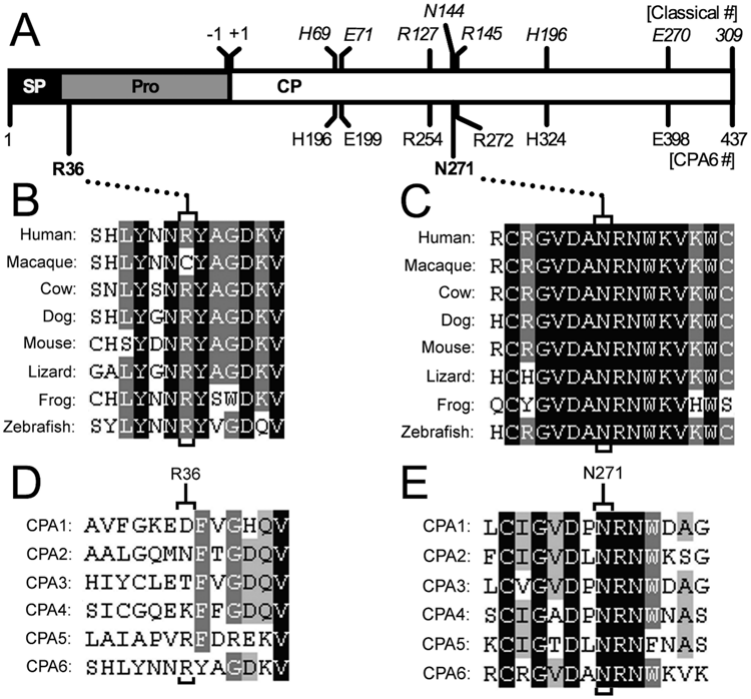
Alignments of Arg36 and Asn271 residues in proCPA6 and related enzymes. (A) Human *CPA6* is translated as a 437 amino acid protein that includes a signal peptide (SP) and a prodomain (Pro). The prodomain, containing Arg36His, acts as an intramolecular chaperone and also maintains the enzyme in an inactive state. The mature enzyme is secreted, and contains only the carboxypeptidase (CP) domain. Carboxypeptidases in the M14 family have several conserved residues, often referred to by their position in the classical numbering system (italics, top numbers), which is based on the active form of CPA1. The residue number in the preproCPA6 sequence are also indicated (bottom numbers), which begins with the translation-initiating methionine. Several critical active site residues are shown. These residues are important for coordination of the zinc (*His69* = His196, *Glu71* = Glu199, *His196* = His324); substrate binding (*Arg127* = Arg254, *Asn144* = Asn271, *Arg145* = Arg272); and catalysis (*Glu270* = Glu398). (B) Clustal Omega was used for multiple sequence alignments to analyze mutated residues. Arg36 is conserved in most proCPA6 orthologs, with the exception of several primates, which have a cysteine at this residue. (C) Asn271 and its neighboring residues are 100% conserved from humans to zebrafish. (D) Across CPA-like family members, Arg36 is not conserved, and is in a region with few conserved residues. (E) Asn271 is conserved in every CPA-like enzyme in humans, as are several neighboring residues.

Each mutation was visualized within the predicted structure of proCPA6 (Fig 2A). The Arg36His mutation occurs at the N-terminus of the prodomain, and is not present in the active mature peptidase. The Asn271Ser mutation occurs within the carboxypeptidase domain, one residue downstream of active site residue Arg272—this corresponds to Arg145 in the classical numbering scheme based on the active form of bovine CPA1. Arg145 is a critical residue in all CPA/B family members involved in binding to the carboxylate group on the C-terminus of the substrate. Asn271 (corresponding to Asn144 in the classical nomenclature) has also been described as a critical active site residue involved in substrate binding [38,39]. Protein sequence alignments of proCPA6 were generated to compare species throughout vertebrate evolution. The Arg36His mutation occurs at a residue that is generally conserved throughout evolution with the exception of some primates, including macaque (*M*. *mulatta*)(Fig 2B). Asn271Ser mutation occurs within a region of very high conservation, and is conserved in all species examined (Fig 2C). Next, we examined conservation among human sequences for CPA subfamily members. Only CPA5 and CPA6 have an arginine at the position corresponding to Arg36 in CPA6, and this region is not highly conserved across subfamily members (Fig 2D). In contrast, Asn271 is strictly conserved among subfamily members, as are the two downstream residues (Fig 2E).

**Fig 3:**
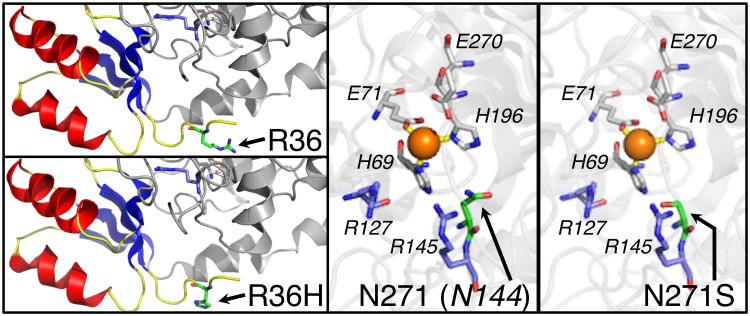
Modeling of Arg36His and Asn271Ser mutations within proCPA6 protein structure. The structure of proCPA6 was modeled based on the crystal structure of related family members as described previously [30]. PreProCPA6 contains a signal peptide, which is removed during synthesis of the protein. Once the signal peptide is removed, Arg36 (green sticks) is one of the most N-terminal residues of the proenzyme, and is located within the prodomain (blue beta sheets, yellow loops, red alpha helices). Arg36 is part of a loop with no known function. Its charge is outward-facing, and not predicted to interact with nearby residues. Asn271 (green sticks) is located within the active site of the enzyme. It is known as *Asn144* in the classical numbering system and is a critical active site residue involved in substrate binding. It faces inwards towards the other residues that participate in substrate binding (blue sticks), zinc-coordination (white sticks) and catalysis (white sticks).

The structure of CPA6 has been modeled based on the crystal structures of related metallocarboxypeptidases: CPA1, CPA2, CPA4, CPB1 and CPB2 [30]. Mutations identified in JME patients were visualized within the structural model. Arg36 is an outward-facing residue near the N-terminus of the prodomain (Fig 3). By contrast, Asn271 faces inwards towards the active site, and is adjacent to a critical residue involved in substrate binding (Fig 3). From this analysis, we predicted that substitution of Ser for Asn271 could potentially change the electrostatic interior of the substrate-binding pocket and render the enzyme inactive. Because Arg36 is present in the prodomain, the Arg36His mutation could only affect the mature form of the enzyme if it affected folding, secretion, or conversion of proCPA6 into CPA6.

**Fig 4:**
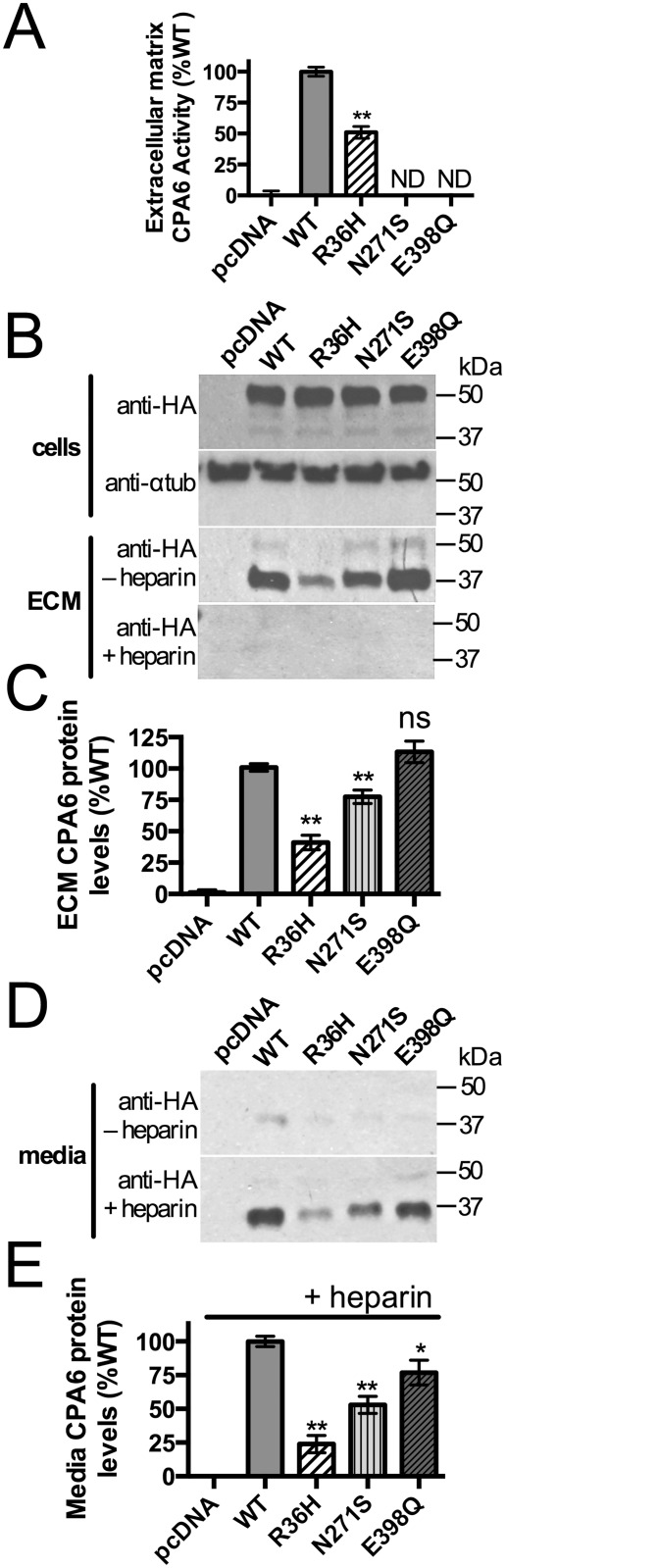
Biochemical analyses of Arg36His and Asn271Ser mutant proCPA6. Wild-type (WT) and mutant proCPA6 tagged on the C-terminus with the HA epitope and His6 sequence (HAH6) were expressed in HEK293T cells by transient transfection. (A) Catalytic activity of ECM-bound CPA6 was assayed with the substrate 3-(2-furyl)-acryloyl-Phe-Phe. The Arg36His mutant proCPA6 produced CPA6 with approximately 50% activity relative to WT, while the Asn271Ser mutant proCPA6 had no detectable activity. Empty vector (pcDNA 3.1) and Glu398Gln proCPA6, an active site mutation, were included as negative controls. (B,C) Western blot analysis of CPA6 in cells and ECM, detected using an anti-HA antibody. All proCPA6 constructs express at equal levels in the cells. Alpha-tubulin immunoreactivity was used as a loading control. ECM levels of CPA6 produced from mutant Arg36His-proCPA6 were ~50% of WT while Asn271Ser CPA6 was expressed at ~75% of WT. The addition of heparin greatly reduced the level of CPA6 in the ECM. (D,E) Analysis of media. Under basal conditions, the level of CPA6 in medium was low, and this was increased by the presence of 400 μg/ml heparin in the culture medium. Levels of the Arg36His and Asn271Ser forms were reduced in the heparin-treated media relative to WT. Abbreviations: ND, not detectable; ns, not significant; *, p<0.05; **, p<0.01 relative to the WT control. Error bars indicate standard error of the mean in panels A (n = 10), C (n≥7), and E (n = 7).

Biochemical analysis of the mutant forms of proCPA6 expressed in HEK293T cells was performed to test functional consequences of the mutations. HEK293T cells were transfected with wild type (WT) or mutant proCPA6, and ECM from these cells was assayed for CPA6 activity. The Arg36His mutation occurs within the prodomain, which is not present in active ECM-bound CPA6. ECM samples from cells transfected with Arg36His proCPA6 showed ~50% CPA6 activity, while those from cells transfected with Asn271Ser proCPA6 had no detectable activity (Fig 4A). Glu398Gln proCPA6, an active site mutant not found in humans, was transfected as a negative control—this mutation eliminates CPA6 activity due to loss of the critical Glu residue required for catalytic activity [22]. The same samples used for assaying CPA6 activity were analyzed by western blotting to quantify protein levels of proCPA6 in cells and CPA6 in the ECM. ProCPA6 levels in the cell lysates were approximately equal between WT proCPA6 and mutant proCPA6 (Fig 4B). In the ECM, however, CPA6 produced from Arg36His proCPA6 was expressed at approximately half the levels as WT CPA6, and Asn271Ser CPA6 was reduced by a small but significant amount (Fig 4B and 4C). When CPA6 activity resulting from Arg36His proCPA6 was normalized to protein expression, it was fully active per unit protein. To examine whether the reduction in CPA6 expression in the ECM could be due to failure to bind to the ECM, media samples were analyzed. For both WT and mutants, only low levels of CPA6 were detected (Fig 4D). Cells grown in the presence of heparin, which causes CPA6 to become soluble, were used as a positive control to ensure that CPA6 could be detected when it is present in the media (Fig 4D and 4E). The pattern of expression was similar between ECM under basal conditions and heparin-treated media, indicating that CPA6 mutants are able to bind to the ECM.

**Fig 5:**
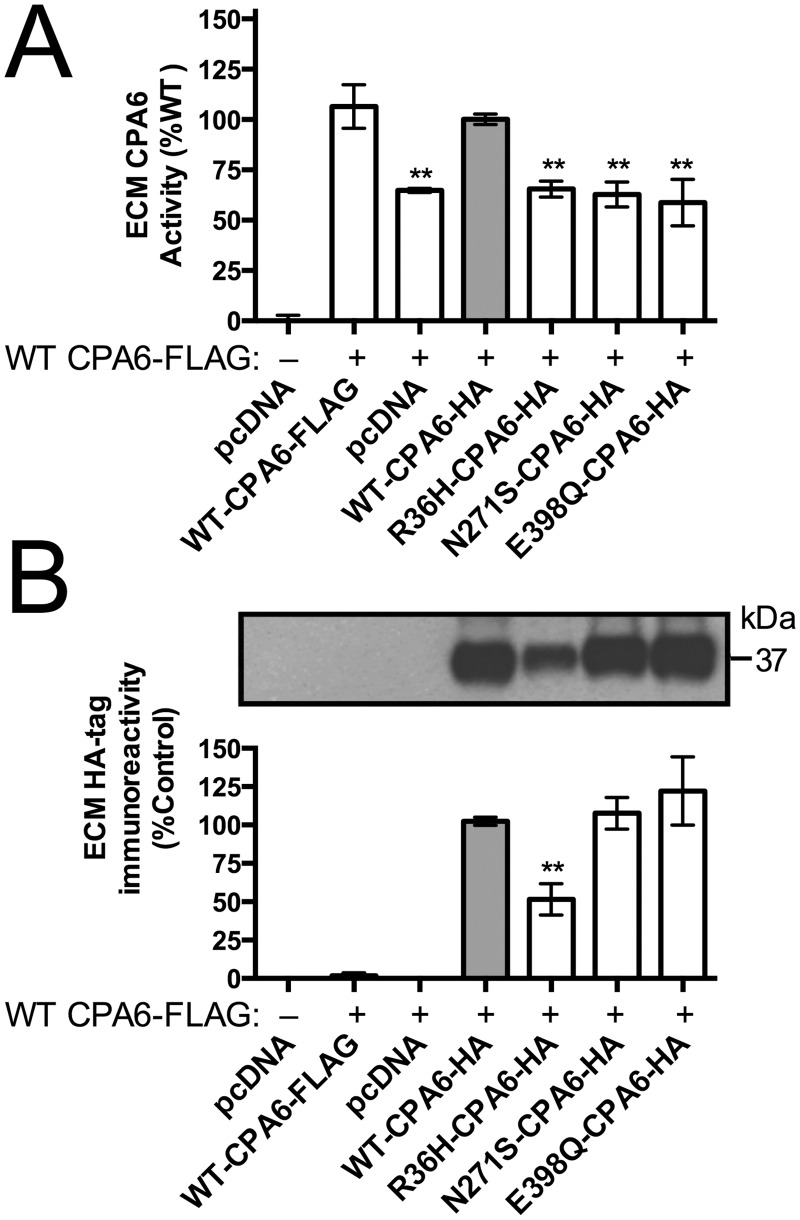
Co-expression of WT and mutant proCPA6 in HEK293T cells. HAH6-tagged or FLAG-tagged wild-type (WT) and HAH6-tagged mutant proCPA6 were co-expressed in HEK293T cells by transient transfection and ECM was analyzed. (A) Enzyme activity of CPA6 in the ECM was assayed using 3-(2-furyl)-acryloyl-Phe-Phe. In co-expression experiments, approximately 55–65% activity was seen when WT-CPA6-FLAG was coexpressed with either of the two inactive mutants (Asn271Ser and Glu398Gln), with the Arg36His mutant, or with empty pcDNA vector; the difference in means between these 4 groups was not statistically significant. (B) HA-tag immunoreactivity in the ECM, measured by western blotting, showed a 50% reduction for Arg36His-CPA6 while levels were unchanged for Asn271Ser and Glu398Gln mutants. Error bars show standard error of the mean in panels A (n≥7) and B (n≥4). **, p<0.01.

Patients harboring *CPA6* mutations are heterozygotes, expressing one normal and one mutant copy. We therefore wanted to test for any interactions between WT and mutant CPA6 by co-expressing WT and mutant protein in the same cells. Cells expressing proCPA6 with a FLAG tag (WT CPA6-FLAG) or with a mixture of FLAG and HA tagged WT CPA6 showed no significant difference in activity. WT CPA6-HA co-expressed with WT CPA6-FLAG was used to normalize data points to 100%. CPA6-FLAG co-expressed with inactive mutants (Asn271Ser or Glu398Gln) or pcDNA 3.1 empty vector showed no significant difference from CPA6-FLAG co-expressed with Arg36His CPA6-HA, with means ranging from ~55–65% (Fig 5A). ECM was collected and analyzed by SDS-PAGE and probed with an anti-HA antibody. WT, Asn271Ser or Glu398Gln CPA6 were present in the ECM at approximately equal levels, while CPA6 resulting from Arg36His-proCPA6 was present at approximately 50% the levels of WT CPA6 (Fig 5B). Because the levels of ECM CPA6 activity were not reduced below the 50% value, the mutants do not appear to have dominant negative effects on WT CPA6.

**Fig 6:**
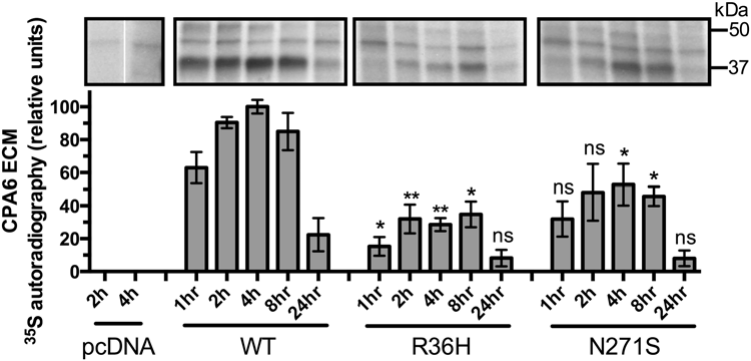
Pulse-chase analysis of HEK293T cells transfected with WT or mutant proCPA6. Cells were labeled with ^35^S methionine and cysteine for 20 minutes (pulse), washed once, and incubated for the time indicated (chase) in media containing non-radioactive methionine and cysteine. ECM was extracted with SDS and analyzed on a denaturing polyacrylamide gel, which was dried and exposed to film for 7–10 days. CPA6, which runs ~37 kDa, was not detected in the cells transfected with pcDNA vector alone. Arg36His samples showed reduced levels of CPA6 relative to WT at all time points, which was statistically significant at all time points except 24h. Asn271Ser samples showed a trend towards reduced levels of CPA6 at all time points, with significant reductions in CPA6 levels at the 4 and 8 hour time points. Comparisons were performed across CPA6 variants for each time point; ns, not significant; *, p<0.05; **, p<0.01 relative to the same time point for the WT sample. Error bars show standard error of the mean (n = 3).

Previous examinations of CPA6 in the ECM have focused on steady state levels at equilibrium. To examine the rate at which CPA6 is deposited into the ECM and degraded from the ECM, cells expressing proCPA6 were incubated with ^35^S methionine and cysteine for 20 minutes (pulse) to allow incorporation of the labeled amino acids, followed by 1–24 hours incubation (chase) with media containing unlabeled methionine and cysteine. ECM samples were collected at the end of the indicated chase time and analyzed by SDS-PAGE. CPA6 represents the most prevalent ^35^S-labeled protein in the SDS extracts of ECM from HEK293T cells transfected with *CPA6* plasmid (Fig 6), although other methods of ECM extraction did not show CPA6 to be a major band (data not shown). Blank vector alone showed only faint bands between 37 and 50 kDa, and no band at 37 kDa where mature CPA6 is detected when cells are transfected with CPA6-expressing plasmid (Fig 6). This indicates that CPA6 can be reliably detected in the ECM by this method (Fig 6). WT-CPA6 showed a similar time course in the ECM as has been previously found [28], with peak levels between 2 and 8 hours (Fig 6). For each chase time, WT CPA6 levels were compared to CPA6 resulting from Arg36His proCPA6 levels. For every time point examined, CPA6 produced from Arg36His proCPA6 showed reduced levels of radio-labeled CPA6. The overall pattern of accumulation and depletion of CPA6 produced from Arg36His proCPA6 in the ECM was similar to that found in WT, with peak levels between 2 and 8 hours (Fig 6, middle panel). The Asn271Ser mutant also showed a similar peak for accumulation in the ECM (Fig 6, right panel).

## Discussion

Following our initial discovery of *CPA6* SNPs as risk alleles for familial FS and TLE [22], as well as for sporadic cases of TLE [22,23], we searched for *CPA6* mutations in another form of epilepsy. In the present study we report two additional *CPA6* mutations in JME patients who also presented with generalized epilepsy. *CPA6* is the second gene related to JME that is not an ion channel or receptor. The only other known non-channel, non-receptor epilepsy gene related to JME is *EFHC1*/*Myoclonin1*, mutations in which have been reported in 3 to 9% of JME patients worldwide [13]. Although myoclonin 1 is not a channel, it is thought to interact with the calcium channel Ca_v_2.3. A mutation in *EFHC1* was also found in a TLE individual [21]. Mutations in both the *EFHC1* and *CPA6* genes have been found in patients with generalized and partial epilepsies.

The two female JME patients who show *CPA6* mutations are currently in remission. It is plausible to hypothesize that CPA6 defects are more involved in the onset of epileptic syndromes than in disease severity. This fits with previous evidence, considering that affected individuals from a single family harboring the same *CPA6* mutation resulted in some family members having a mild phenotype (simple FS) and others with severe clinical features (TLE and complex FS) [22]. Similar to the present findings, a recent study has identified a family in which multiple members harbor the same missense mutation in *CASR*, which encodes an extracellular calcium sensing receptor. Members of this family present with several different seizure disorders including JME, TLE and FS [9]. We hypothesize that *CPA6* mutations are part of a complex pattern of inheritance that predisposes towards seizures and epilepsy in general, but not necessarily a specific subtype of epilepsy.

Establishing causality between mutations and disease presents difficulties in diseases with complex patterns of inheritance. In a given patient, multiple point mutations may contribute to the disease state, and mutations that increase the likelihood of seizures may not have high penetrance.

*CPA6* mutations found in JME patients impact the protein, showing that they are functional. Modeling point mutations within the predicted structure revealed that the Arg36His mutation occurs at the very N-terminus of the prodomain of the enzyme just downstream of where the signal peptide is removed. It is outward-facing and is not nearby to other residues in the model of the structure. This structural model, which is based on solved structures of other related enzymes [30] may be inaccurate at the N-terminal region because of lack of recognizable motifs and variation in the lengths of this region among different CPA family members. Arg36 of proCPA6 is generally conserved among species except in some primates where it is a cysteine. Because of these factors, it is difficult to predict the effect of this substitution. In contrast, the Asn271Ser mutation is within the carboxypeptidase domain and is highly conserved throughout all members of the carboxypeptidase family. Asn271 corresponds to Asn144 using the classical numbering system of carboxypeptidases, and in other enzymes this residue has been described as a critical active site residue. The finding that the Asn271Ser mutation results in inactive CPA6 fits with our prediction that this mutation would be deleterious. These findings are consistent with a mutation previously described in TLE patients (His196Arg), which results in the replacement of a critical zinc-coordinating histidine with a residue that cannot coordinate with zinc [23] and similarly results in approximately normal levels of protein in the ECM but no detectable enzyme activity. These findings indicate that the Asn271Ser mutant is folded, processed, secreted, and can bind to the ECM, but it is not able to cleave substrates.

Previously, coexpression was used to show that WT and mutant CPA6 do not interact when co-expressed in HEK293T cells [23]. Similarly, co-expression experiments in the present study showed no dramatic interaction between WT and the two new mutant CPA6 forms identified in JME patients. Interestingly, ECM from cells expressing Arg36His proCPA6 had approximately equal activity to ECM from cells co-expressing WT and catalytically inactive proCPA6.

Pulse-chase analysis with radio-labeled amino acids is a useful technique to investigate the rate of protein synthesis and secretion, and has been used to study other metallocarboxypeptidases in the same family [40–43]. Using this technique, we found that CPA6 from the mutant Arg36His proCPA6 and Asn271Ser proCPA6 reach their maximum protein levels in the ECM at essentially the same rate as WT CPA6 although the maximal level is reduced, consistent with the results from western blotting. In the case of the Asn271Ser mutant, this may reflect a partial defect in the folding of the mutant enzyme. The Arg36His mutant is more complicated because the mutation is within the pro domain, and this region is cleaved from CPA6 prior to deposition within the ECM. Thus, the amino acid sequence of CPA6 is identical for the protein produced from WT proCPA6 and Arg36His proCPA6. However, the abnormal pro region of the Arg36His proCPA6 can affect folding or conversion of proCPA6 into CPA6, and the results from the pulse-chase studies support these possibilities.

While the mechanism by which *CPA6* mutations predispose patients to epilepsy remain unclear, several studies have explored functions of other proteins mutated in TLE and JME patients. EFHC1/Myoclonin1 is thought to be involved in mechanisms for removing unnecessary neurons during brain development by coordinating cell death. Loss of function mutations in this protein are thought to decrease apoptotic events that lead to an increase in density of neurons and hyperexcitability, which may be a causal factor in the genesis of epilepsies [12,44]. Overexpression of myoclonin1 in mouse primary hippocampal cultures has been shown to cause apoptosis, which can be partially reversed by an antagonist acting at the Ca_v_2.3 channel [12]. Myoclonin1 has also been studied for its role in mitotic spindle organization and interactions with microtubules [14,45]. Taken together, there is substantial evidence for the role of EFHC1 in brain development, and consequently for the role of developmental defects in JME pathogenesis. Previously, epilepsy patients with mutations in *CPA6* have been reported with brain malformations, intellectual disability and hippocampal sclerosis, suggesting that CPA6 is involved in brain development [22,23]. Further studies of humans or animals with mutations in *CPA6* may uncover further evidence for the role of this enzyme in development. CPA6 has been shown to cleave neurotensin *in vitro*, as well as several other peptides [28]. It is possible that altered signaling of these and other peptides affects neuronal excitability or brain development, leading to an increased likelihood of seizures.

While the role of CPA6 in epileptogenesis is unclear, it is likely that it synthesizes, modulates or degrades peptides and protein signaling molecules involved in epileptogenesis. If this signaling is altered in the absence or reduction of CPA6 activity, it could lead to altered development of the nervous system or altered firing in neural networks [46]. CPA6 is highly conserved and localizes to specific brain regions over the course of development [31,47]. While its substrate specificity is highly similar to other CPA-like enzymes in the same family [48], CPA6 is the only member with aliphatic/aromatic substrate specificity that binds tightly to the ECM [30]. These characteristics point to a unique function of CPA6. Interestingly, alterations in *CPA6* have been identified in other human diseases unrelated to epilepsy [49,50], although it remains unclear if there is any causal relationship [47]. Clarification of the involvement of CPA6 in these other diseases may provide substrate candidates that may be similar to its substrates in the nervous system.

While a great deal of research has emphasized the importance of endopeptidases in peptide processing, it has long been known exopeptidase processing is also required for the generation of most active peptide signaling molecules [51,52]. Further, C-terminal proteolysis is necessary in a variety of different biological contexts, and is one mechanism by which full-length proteins such as fibrin [53], tubulin [54–56] and Wnt4 [57] are modulated. The exact substrates of CPA6 remain unknown, and further studies are needed to identify CPA6 substrates that explain how *CPA6* mutations increase the likelihood of developing epilepsy. Such studies may uncover novel pathways by which epilepsy develops and/or progresses, and lead to the identification of useful targets for intervention.

## Supporting Information

S1 Table Common CPA6 variants in JME patients. The common CPA6 variants are rs10957393 (c.133 T>C, p.Phe45Leu); rs17853192 (c.518 C>G, p.Ser173Cys); and rs17343819 (c.746 A>G, p.Asn249Ser). All of these have a minor allele frequency ≥0.08. None of these are more frequently observed in the JME patients, compared to the control group in our analysis.
(PDF)
