# Supplementary material for: Novel Carboxypeptidase A6 (CPA6) Mutations Identified in Patients with Juvenile Myoclonic and Generalized Epilepsy
Source: PLoS One. 2015 Apr 13;10(4):e0123180. doi: 10.1371/journal.pone.0123180 (PMC4395397; doi:10.1371/journal.pone.0123180)
Supplement: S1 Table — The common CPA6 variants are rs10957393 (c.133 T>C, p.Phe45Leu); rs17853192 (c.518 C>G, p.Ser173Cys); and rs17343819 (c.746 A>G, p.Asn249Ser). All of these have a minor allele frequency ≥0.08. None of these are more frequently observed in the JME patients, compared to the control group in our analysis. (PDF) [file pone.0123180.s001.pdf]

Supplementary Table S1. Common *CPA6* variants in JME patients.

	Control n (%)		JME n (%)	
**rs10957393 (c.133 T>C, p.Phe45Leu) MAF: C=0.2322**				
Allele	476		248	
T	394	(82.8)	206	(83.1)
C	82	(17.2)	42	(16.9)
p_u				*0.921*
Genotype	238		124	
TT	156	(65.5)	82	(66.1)
CT	82	(34.5)	42	(33.9)
CC	0	(0.0)	0	(0.0)
p_u				*0.912*
**rs17853192 (c.518 C>G, p.Ser173Cys) MAF: G = 0.0891**				
Allele	474		243	
C	439	(92.6)	230	(93.5)
G	35	(7.4)	16	(6.5)
p_u				*0.661*
Genotype	237		123	
CC	202	(85.2)	107	(87.0)
CG	35	(14.8)	16	(13.0)
GG	0	(0.0)	0	(0.0)
p_u				*0.999*
**rs17343819 (c.746 A>G, p.Asn249Ser) MAF: G = 0.0791**				
Allele	476		248	
A	411	(86.3)	201	(83.0)
G	65	(13.7)	41	(17.0)
p_u				*0.245*
Genotype	238		121	
AA	176	(74.0)	82	(67.8)
AG	59	(24.8)	37	(30.6)
GG	3	(1.2)	2	(1.6)
p_u				*0.767*

p_u = uncorrected *p*-value
MAF = minor allele frequency (source: 1000 genomes, accessed Oct 2014)
